# Effect of Cu Ions Implantation on Structural, Electronic, Optical and Dielectric Properties of Polymethyl Methacrylate (PMMA)

**DOI:** 10.3390/polym13060973

**Published:** 2021-03-22

**Authors:** Athar N. Akhtar, G. Murtaza, M. Ahsan Shafique, Ahmed S. Haidyrah

**Affiliations:** 1 Centre for Advanced Studies in Physics, GC University, Lahore 54000, Pakistan; atharnaeemgcu@gmail.com (A.N.A.); muhammadahsan@gcu.edu.pk (M.A.S.); 2 Nuclear and Radiological Control Unit, King Abdulaziz City for Science and Technology (KACST), Riyadh 11442, Saudi Arabia; ahydrah@kacst.edu.sa

**Keywords:** PMMA, Cu implantation, optical properties, optoelectronic applications

## Abstract

In this work, the effect of ion bombardment on the optical properties of Polymethylmethacrylate (PMMA) was studied. Polymer samples were implanted with 500 keV Cu^+^ ions with a fluence ranging from 1 × 10^12^ to 1 × 10^14^ ions/cm^2^. X-ray Diffractometer (XRD) study indicated a relatively lower variation with a higher dose of ions. Fourier Transform Infrared (FTIR) spectra exhibited that with the implantation of Cu ions the intensity of existing bands decreases, while the result confirms the existence of a C=C group. The pristine and ion-implanted samples were also investigated using photoluminescence (PL) and Ultra Violet-Visible (UV-VIS) spectra. The optical band gap (E_g_) was observed up to 3.05 eV for the implanted samples, while the pristine sample exhibited a wide energy-gap up to ~3.9 eV. The change in the optical gap indicated the presence of a gradual phase transition for the polymer blends. The dielectric measurements of the pristine and Cu-implanted PMMA were investigated in the 10 Hz to 2 GHz frequency range. It was found that the implanted samples showed a significant decrease in the value of the dielectric constant. The value of the dielectric constant and dielectric loss of the PMMA and Cu-implanted samples at a 1-kHz frequency were found to be ~300 and 29, respectively. The modification of the PMMA energy bandgap in the current research suggested the potential use of Cu implanted PMMA in the field of optical communications and flexible electronic devices.

## 1. Introduction

Polymers with large molecular weights are promising candidates due to their versatile properties, forms and compositions. The range of applications varies, e.g., biomedical, electronic, structural and optical fields, which leads to the fabrication of medical implants, microelectronics, parts for the automotive industry, antireflective coatings and optical sensors in this modern age [1,2,3,4]. These applications are the consequence of polymers’ inherent and unique properties, such as being anti-corrosive, lightweight, economic, moldability, optical clarity, weather-resistive and ease of availability [5,6]. Functional desire and the enhancement of such applications can be easily fulfilled by introducing modification in the chemical and structural properties of polymers. Modification techniques that can be helpful in order to enhance the various properties of polymers are gamma rays, ultraviolet, neutron, electron and ion implantation. Different polymers, such as Polyethene oxide (PEO), low-density polyethene (LDPE), Poly vinyl Alcohol (PVA), Polytetraflouro ethylene (PTFE), Poly vinyl chloride (PVC), Polyethene glycol (PEG), Polycarbonate (PC), Polypropylene (PP), Polyallyldiglycol carbonate (PADC) and Polymethylmethacrylate (PMMA) exhibit a remarkable chemical and physical properties after surface modification [7]. Among these polymers, PMMA is extensively used by researchers due to its excellent properties, such as chemically inertness, better electrical insulation, excellent optical transparency and low wettability. PMMA is considered preferable as an alternate material to glass and different optical lenses [8]. Different techniques are used by researchers to alter the structural and chemical properties, such as heat treatment, doping, change in composition, mixing of ion beams, ion trapping and ion beam modification. An important and widely used techniques is the modification of surface properties of polymers using electrons, neutrons, ion beams and gamma rays for versatile applications of polymers in background radiation devices, such as ballistic capsule, nuclear reactor, sterilization irradiators and high energy particle accelerators [9,10]. This technique causes a transformation in the chemical composition and structure with a well-controlled method [11]. The PMMA structure is strongly sensitive to the irradiations of energetic ions [12,13,14,15,16]. However, such surface properties are correlated with ion implantation parameters, such as ion impact, the mass of the ion and the density of ion. The energetic ions in this process interact with the target leading to various phenomena, such as chain scissoring, electron excitation, ionization of atoms, the formation of free radicals, emission of volatile gases and the formation of multiple carbon–carbon bonds [17,18,19]. Ion implantation generates irreversible structural changes required for the production of advance polymers owing to the modified attributes. The irreversible changes effectively depend on the energy loss of implanted ions, range of ion penetration in sample matrix and ion implantation conditions, i.e., energy, skin depth and radiant ion dose [20,21,22,23,24]. The modification made by ion implantation is the outcome of the energy loss of incident ions with the target sample. The interaction of incident ions with target causes mainly two types of processes: (1) nuclear stopping and (2) electronic stopping [25]. In the nuclear stopping mechanism, momentum is transferred from the ion to the matrix of the host material by elastic collision. In electronic stopping, glancing inelastic collision occur during the interaction of incident ions with a target. The electronic stopping process produces excitation in the target orbital electrons from a low energy level to a high energy level and culminating in the ionization of atoms [26,27,28]. The loss in total energy is due to power losses by nuclear stopping (Sn) and electronic stopping (Se) powers, respectively. These two stopping powers are normally responsible for producing cross-linking and chain scissoring in polymeric materials. Cross-linking is mainly observed in the electronic energy loss of ions in the polymers and chain scissoring activity is predominantly governed by the nuclear energy loss process. The ratio (Sn/Se) in the total energy loss gives the probability of structural modification of polymers induced by ion implantation [29,30,31].

In the present study, the Pelletron accelerator was employed to modify the physical properties of polymeric materials without changing their bulk properties. The implantation process offered improvements/alterations in their properties, e.g., electrical, optical, thermoelectric, surface morphology, mechanical, etc. Our main objective was to enhance the electrical properties of PMMA polymer by introducing/improving conducting behavior in the insulating polymer via the ion implantation technique. The modification revealed the best aspects of PMMA physical properties. This approach was new and, to the best of our knowledge, there was less data regarding the modification of PMMA electronic and dielectric properties due to the metal ions. Various characterization techniques were used to analyze these materials, like X-ray diffraction (XRD) for structural analysis, UV-visible plus photoluminescence spectrophotometry to investigate the optical behavior and FTIR for the verification of the existence of functional groups. The effect of the interaction of alternating current (AC) signal with the polymer was observed by analyzing dielectric properties.

## 2. Experimental Setup

**Figure 1:**
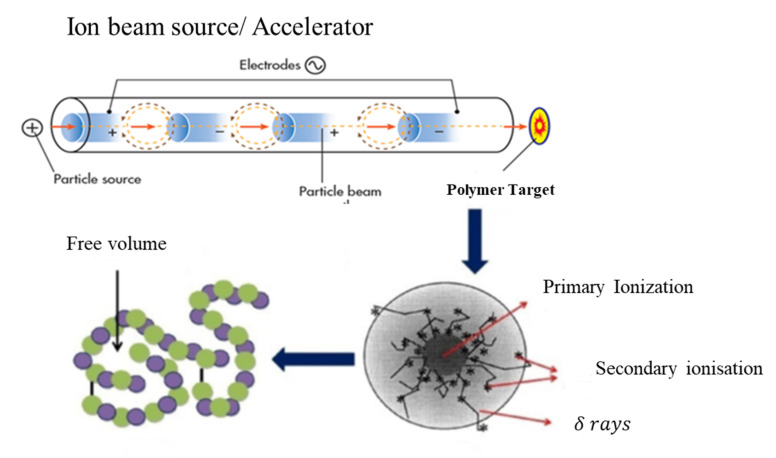
Schematic diagram for ion beam interaction with Polymethylmethacrylate (PMMA) materials.

Cu^1+^ ions beam of 500 KeV energy with a beam size of 2 × 2 cm^2^ from the Pelletron linear accelerator (6SDH-2 NEC, Middleton, WI, USA) installed at the Centre for Advanced Studies in Physics (CASP) GC University Lahore was used to perform the ion beam treatment for the Polymethyl methacrylate (PMMA) targets. Three PMMA targets of 2 × 2 cm^2^ were mounted on the sample holder and exposed to an ion beam with a fluence ranging from 1 × 10^12^ to 1 × 10^14^ ions cm^−2^ and the fourth sample, which was not implanted, was referred to as a pristine sample. A schematic diagram for the complete process of ions with the polymer is shown in Figure 1.

**Figure 2:**
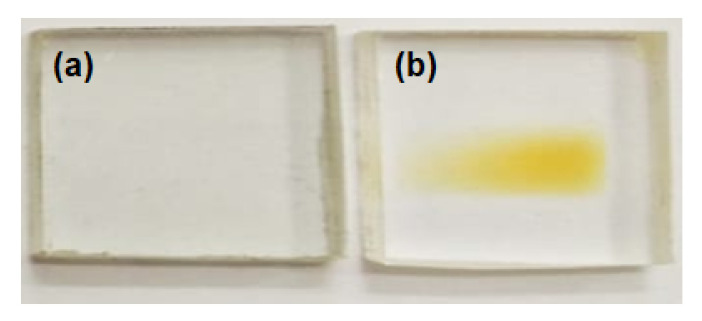
(**a**) Pristine and (**b**) Cu-implanted PMMA sheets with maximum dose of 1 × 10^14^ ions/cm^2^.

The parameters used for the irradiation of samples are presented in Table 1. The ion beam was focused on a spot of ~1 mm in diameter and, to ensure uniform irradiation, the magnetic scanner continuously scanned over the complete area of the samples. Figure 2 depicts the representation of the pristine and high dose surface of the PMMA samples. 

To avoid the channeling effect, implantation was performed at a low angle with respect to the ion beam direction. The electronic energy loss (S_e_) and nuclear energy loss (S_n_) for PMMA samples was calculated using the Stopping and Range of Ions in Matter (SRIM) simulation program [32].

### Characterization

The phase composition and structure of samples were analyzed using X-ray diffraction (XRD) (PANalytical X’Pert Pro X-ray diffractometer, Philips, Almelo, The Netherlands) with a Cu-Kα source (λ = 1.5418 Å). XRD patterns scanning range was 20° to 80° with a step size of 0.05°. Fourier transform infrared (FTIR) spectra were obtained using attenuated total reflection (ATR) (Model IR Prestige-21, SHIMADZU, Kyoto, Japan) to observe the functional groups of the samples with a scan rate 32. The wavenumber and spectral resolution were set in the range of 550 to 4000 cm^−1^ and 4 cm^−1^, respectively. The rate of photogenerated charge carriers recombination was observed using a fluorescence spectrometer (F-4500, HITACHI, Tokyo, Japan) at room temperature. UV–vis spectrophotometer (Genesys 10S spectrophotometer, Thermo Fisher Scientific, Waltham, MA, USA) was used to analyze the absorption behavior. The frequency-dependent dielectric characteristics were analyzed using 6500B Wayne Kerr Impedance Analyzer (Wayne Kerr Electronics, Bognor Regis, West Sussex, UK). These parameters, i.e., tangent loss (tan δ) and frequency-dependent dielectric constant (ε) were calculated for a specimen having a capacitance C, diameters d and area was represented by A. 

## 3. Results and Discussion

### 3.1. SRIM Analysis

**Figure 3:**
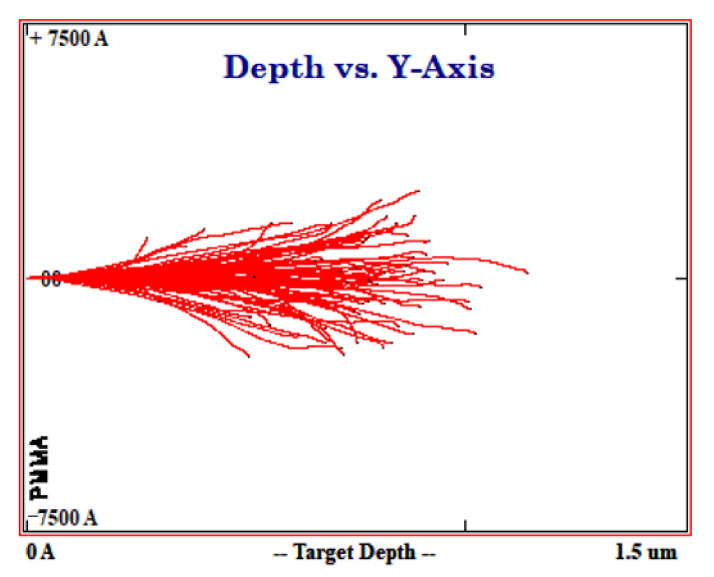
Penetration depth profile of Cu ions, as a function of the implant energy using the Monte Carlo SRIM code.

The simulation results are shown in Figure 3. The results showed a peak at the depth of 60 nm representing the implantation profile peak. The study showed that heavy-ion stopping global accuracy of SRIM-2013 simulations was about 6.0 % [33]. This concluded that 500 keV Cu^+^ ions would stop within the ~100 nm thick PMMA layer. 

### 3.2. X-ray Diffractometer Analysis

**Figure 4:**
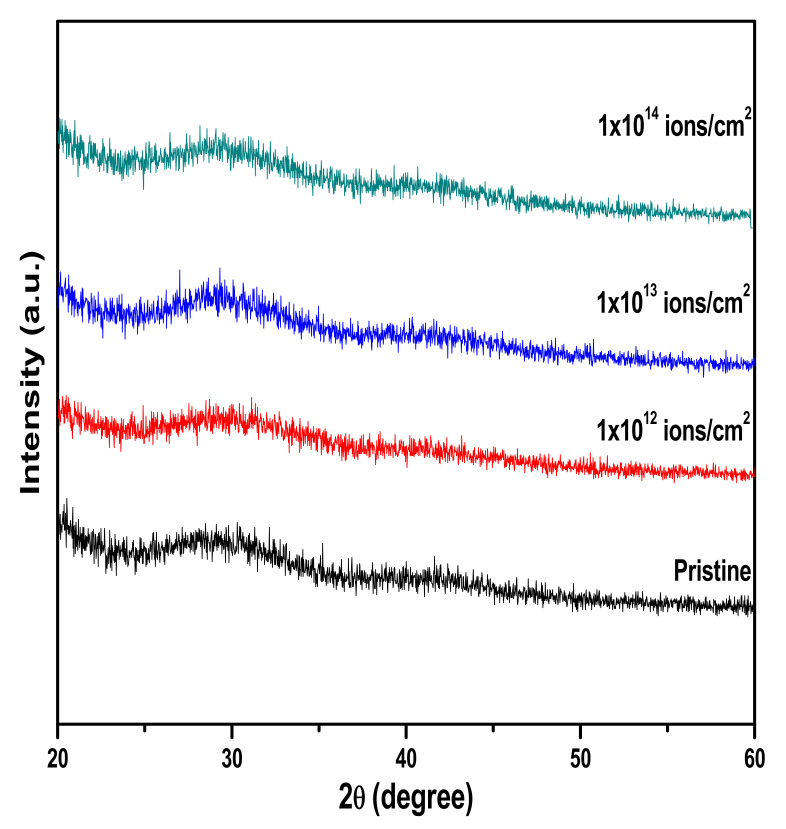
Effect of Cu ion implantation on PMMA using different fluences with a beam energy of 500 KeV.

XRD patterns of pristine and copper irradiated PMMA are shown in Figure 4. Results revealed that broad diffraction at 2θ = 29.74° for unirradiated PMMA indicated the amorphous nature of PMMA. Lovell and Windle [34] observed three broad halos in the PMMA XRD profile. They suggested that the first halos signifies the presence of intermolecular components, while the second and third halos represent intermolecular separation. Weak second and third halos were observed in the treated and pristine samples. Furthermore, the similar size of halos in all the samples was the same, which indicated the nonoccurrence of scission effect by copper implantation.

### 3.3. FTIR Analysis

**Figure 5:**
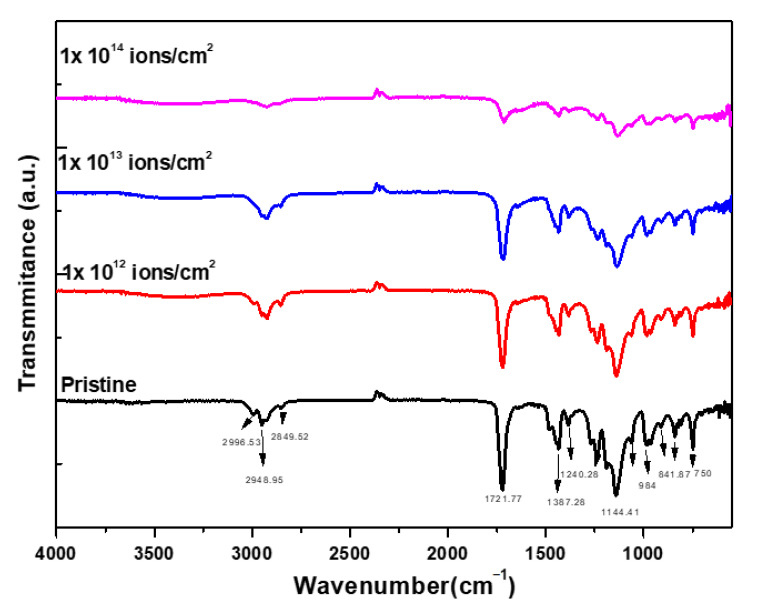
FTIR spectrum of Cu^+^ implanted PMMA for different doses.

The infrared active molecular groups of carbon, oxygen and hydrogen were identified in PMMA, which are presented in Table 2. Certain effects were noticed after ion implantation, such as transmittance being modified due to cross-linking, the chain of polymers being degraded and the formation of a new chemical bond, as shown in Figure 5. The presences of groups of carbonyl were noticed due to the IR peak at 1720 cm^−1^. The peaks in the IR spectrum of both pristine and treated at 1440 and 840 cm^−1^ represented the C-H bending and C-H_2_ rocking vibrations. The peaks in the range of 1270–995 cm^−1^ represented the C-O-C stretching vibrations [35,36].

### 3.4. Photoluminescence and UV-Vis Analysis

**Figure 6:**
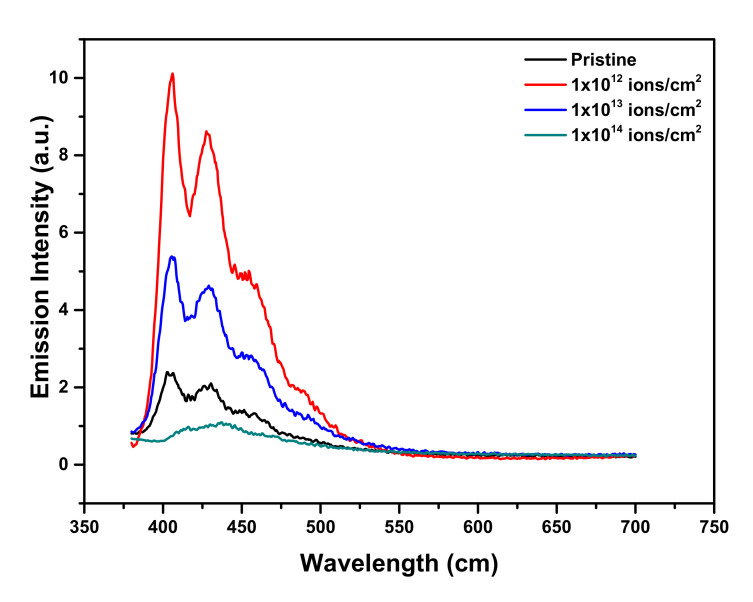
Photoluminescence spectra of pristine and Cu^+^ implanted PMMA.

The pristine and Cu-ion-beam-implanted samples were analyzed using photoluminescence spectra. As shown in Figure 6, it was clear evidence that at certain doses, the obtained intensity in the PL spectra of the implanted PMMA was more intensive than that of unimplanted PMMA. 

**Figure 7:**
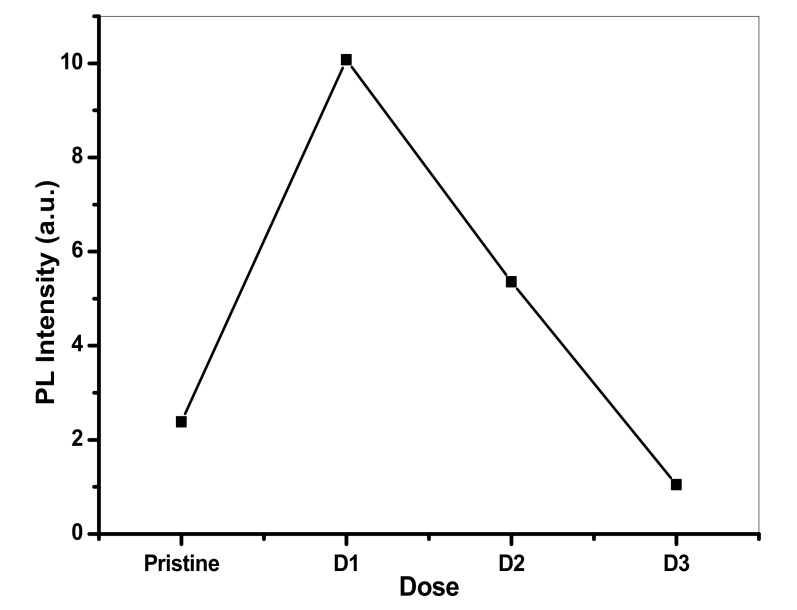
Dose vs. obtained intensity.

Results also revealed that due to Cu^+^ ion implantation, PL emission was present and it appeared to be much more prominent than in the lower energy region. On the other hand, below irradiation at 450 keV Cu^+^, PLE occurred notably at lower doses, particularly much absorbance was noted at 1 × 10^12^ ions/cm^2^. While the further rise in the implantation dose lead to a decrease in the absorbance intensity of the PL spectra, as shown in Figure 7.

**Figure 8:**
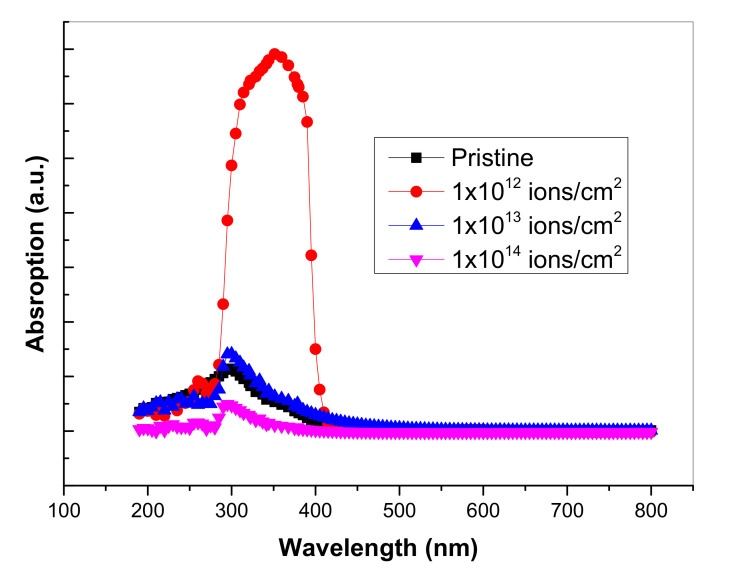
UV-vis absorption spectra for pristine and Cu^+^ implanted samples.

Due to the modification, the broader value of the band of PMMA at ~400 nm showed a comparatively narrow and strong luminescence band at ~405 nm with FWHM ~50 nm. Certain spectral bands were also visible at the values of 427 nm, and 430.15 nm, respectively. These spectral characteristics were retained till the maximum available dose (D = 1 × 10^13^ ions/cm^2^). A logical justification for these spectral improvements in the creation of new states of luminescence and associated transitions was that the Cu^+^ related defect complexes were emanating them [37]. Moreover, the decrease in the absorbance intensity due to the higher dose of Cu ions might have been due to the dissipation of energy which increased the possibility of defects and the consequences of polymer structure degradation. UV-vis spectroscopy was also used to analyze the variation of optical bandgap in the UV-visible spectrum region of ~150 to 800 nm. Figure 8 exhibits the absorption spectra of UV-vis data. It showed that the maximum absorption occurred at the dose (D = 1 × 10^12^ ions/cm^2^). To identify the existence of energy band gap behavior of pristine and implanted PMMA samples, the UV-vis spectra translated into Tauc’s plot used the following equation [38],

(1) $$\alpha \left(\upsilon \right)h\upsilon =B{\left(h\upsilon -E_{g}\right)}^{r}$$

where *α* is the absorption coefficient, *hv* is the incident photon, *E_g_* is the optical band gap and *B* is energy independent constant.

**Figure 9:**
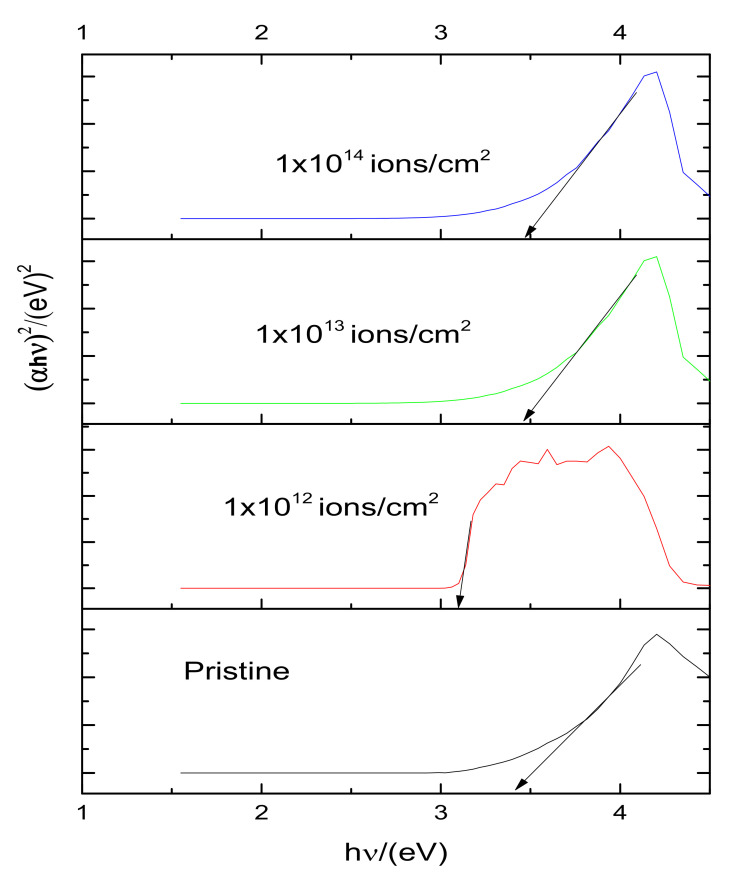
Tauc’s plot for pristine and Cu implanted samples.

Figure 9 reveals that the optical band gap (E_g_) decreased up to 3.05 eV for the implanted samples, while the energy gap in pristine PMMA was usually due to the presence of inactive carbon single bonds. The pristine sample exhibited a wide energy gap of ~3.9 eV as shown in Figure 9. It was observed that ion fluence displaced the ultraviolet wavelength region to the visible blue region. This showed the development of double bonds along the polymeric backbone [39]. The shifted region towards the vis region usually transformed the PMMA color into yellowish, as shown in Figure 2 [40].

### 3.5. Dielectric Measurements

The formula used to obtain the value of the dielectric constant is given as,

*ε_r_* = *cd*/*Aε*_0_ (2)

**Figure 10:**
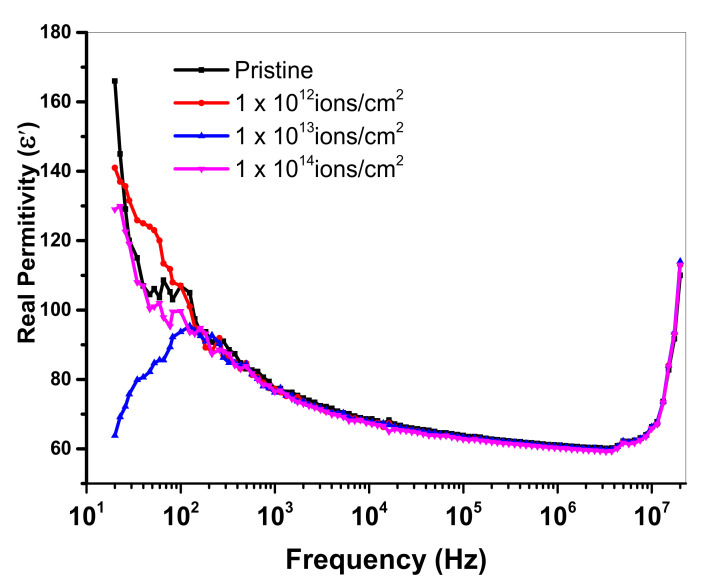
Variation in the real part of dielectric constant (ε′) with frequency.

**Figure 11:**
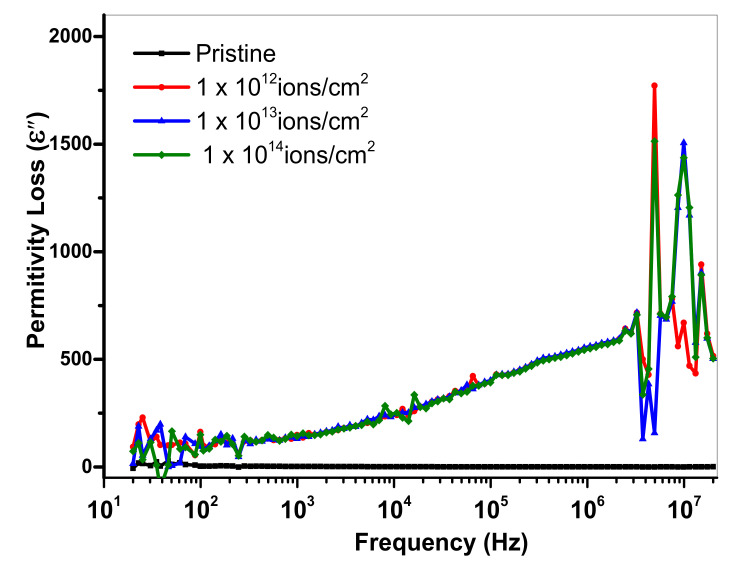
Variation in the real part of dielectric constant (ε′′) with frequency.

**Figure 12:**
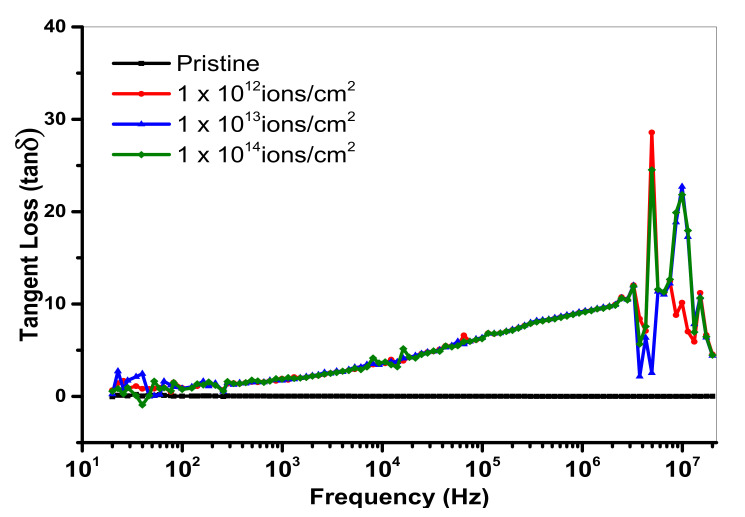
Variation of energy loss of pristine and Cu-ion-implanted samples.

Here, *c* denotes capacitance, *d* represents thickness, *A* is an area and *ε_0_* displays permittivity having worth of 8.854 × 10^−12^ Fm^−1^. Figure 10 and Figure 11 show the dielectric loss and dielectric constant of PMMA at room temperature as a function of frequency. At less than a 1-kHz frequency, when the copper ion was introduced, the value of the dielectric constant of pristine specimen lessened. At a lower frequency, the value 10^13^ ions/cm^2^ represents minor lessening. However, the dielectric constant largely reduced at a higher frequency. The concept of dispersion of polarization along frequency was implemented to demonstrate the falling trend of dielectric constant in the higher frequency region. The dielectric polarization of a particular block was the sum of all the kinds of polarizations, i.e., ionic polarization, electronic polarization and interfacial and dipolar polarization. At the frequency of 1 kHz, Maxwell–Wagner polarization was responsible for the greater values of dielectric constant. Such kind of polarization was originated from the interface of insulator-conductor. The gathering of dipoles or space charges at interfaces resulted in interfacial polarization. Within the low frequency area, space charges had enough interval to respond to the functional field. However, in high frequency area, the applied field was very fast, so that space charges had no time to respond to the applied field and, thus, the polarization effect did not occur [41]. From Figure 10, it is clear that the dielectric loss was reduced with an upturn of frequency. The presence of mobile charges in the polymeric chain at a smaller frequency was the main reason for this variation. As in high frequency, the space charges get it tough to respond to the applied field, so the charged gathering occurred on the behalf of polarization losses at high frequency. Hence, the value of dielectric loss declined [42]. Usually, the dielectric loss factor (tan δ) is considered to estimate the power loss in dielectric materials. For both real and imaginary components of dielectric constant, the dielectric loss tangent was given as: tan δ = ε′′/ε′. The reliance of the dielectric loss factor on frequency is elaborated on in Figure 12. The reduction in the tangent loss with respect to frequency was observed and was in agreement with the literature [43].

### 3.6. AC Conductivity

**Figure 13:**
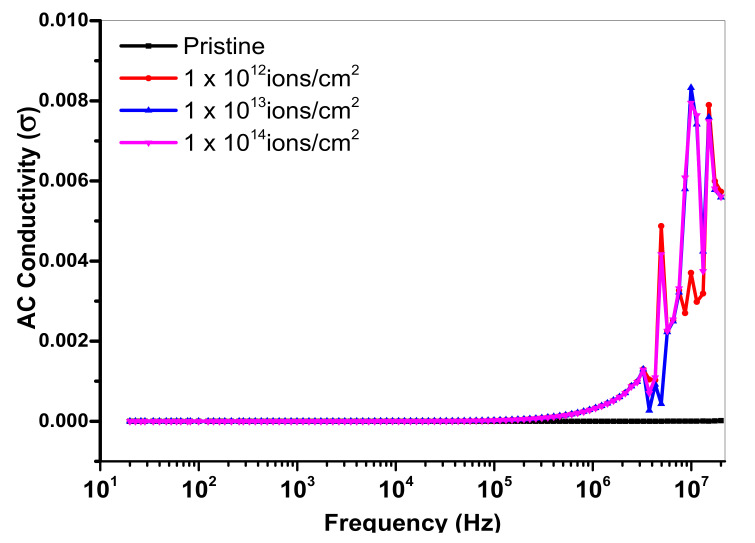
Variation of AC conductivity with different doses of Cu ions.

The frequency dependence of ac-conductivity was calculated using the following relation, $\sigma_{\mathrm{ac}}=2{\pi f\varepsilon }_{0}\varepsilon \prime \prime$, where f is the frequency applied, ε″ is the dielectric loss and ε_o_ is the permittivity of free space. Figure 13 depicts that ac conductivity remained constant for the pristine sample of PMMA while Cu implanted samples showed an increasing trend in the ac conductivity with increasing frequency. The graph in Figure 13 also shows that at higher frequency, PMMA with Cu ions of 5 × 10^13^ ions/cm^2^ showed a sharp increase at a high frequency. Results revealed that at a higher applied frequency and different Cu ion doses, the bonds started to rotate resulting in dielectric transition with accessible flexible polar bonds. This will lead to chemical changes in the polymer chain by the formation of complex charge transfer, which will enhance the AC conductivity [44].

## 4. Conclusions

In this work, a new route was adopted to embed metallic Cu ions in a PMMA matrix to modify the physical properties. PMMA samples were bombarded with 500 keV Cu^+^ ions with fluencies ranging from 1 × 10^12^ to 1 × 10^14^ ions/cm^2^. XRD study indicated relatively less variation with a higher dose of the ions. FTIR spectral study on the implantation of Cu ions revealed the existence of a C=C group and for the different fluence of the copper ions, the intensity of the observed bands decreased. An optical band gap (E_g_) was observed up to 3.9 eV for the pristine samples, while a significant reduction in the bandgap, up to 3.05 eV, was observed using Cu inclusion. The dielectric measurement of the pristine and Cu implanted PMMA revealed that the implanted samples also showed a significant decreased in the value of the dielectric constant. The conclusion drawn from the above investigations suggetsted the fruitful intercession of Cu ions in PMMA to modify the physical properties, which might be suitable for application in optoelectronic devices.

## Figures and Tables

**Table 1:** Fluencies of 500 keV Cu^+1^ ions used for ion implantation.

PMMA Samples	Fluence (ions/cm^2^)
PMMA 1	0
PMMA 2	1 × 10^12^
PMMA 3	1 × 10^13^
PMMA 4	1 × 10^14^

**Table 2:** Position of different bands of the FTIR spectra for pristine and implanted PMMA.

Band Position	Wave Number (cm^−1^)
C-H asymmetric stretching	2996.53
C-H symmetric stretching	2948.95
CH_2_ stretching vibration	2853.07
C=O stretching vibration	1721.77
C-H bending vibration	1387.28
C-O stretching vibration	1240.28
C-O-CH_3_	9984.00
O-CH_3_	901.53
CH_2_	841.87
C-C	750.26

## Data Availability

Not applicable.
